# Supplementary material for: Engineering of an electrically charged hydrogel implanted into a traumatic brain injury model for stepwise neuronal tissue reconstruction
Source: Sci Rep. 2023 Feb 14;13:2233. doi: 10.1038/s41598-023-28870-z (PMC9929269; doi:10.1038/s41598-023-28870-z)
Supplement: Supplementary file 1 — Supplementary Legends. [file 41598_2023_28870_MOESM1_ESM.pdf]

Supplementary Figure 1

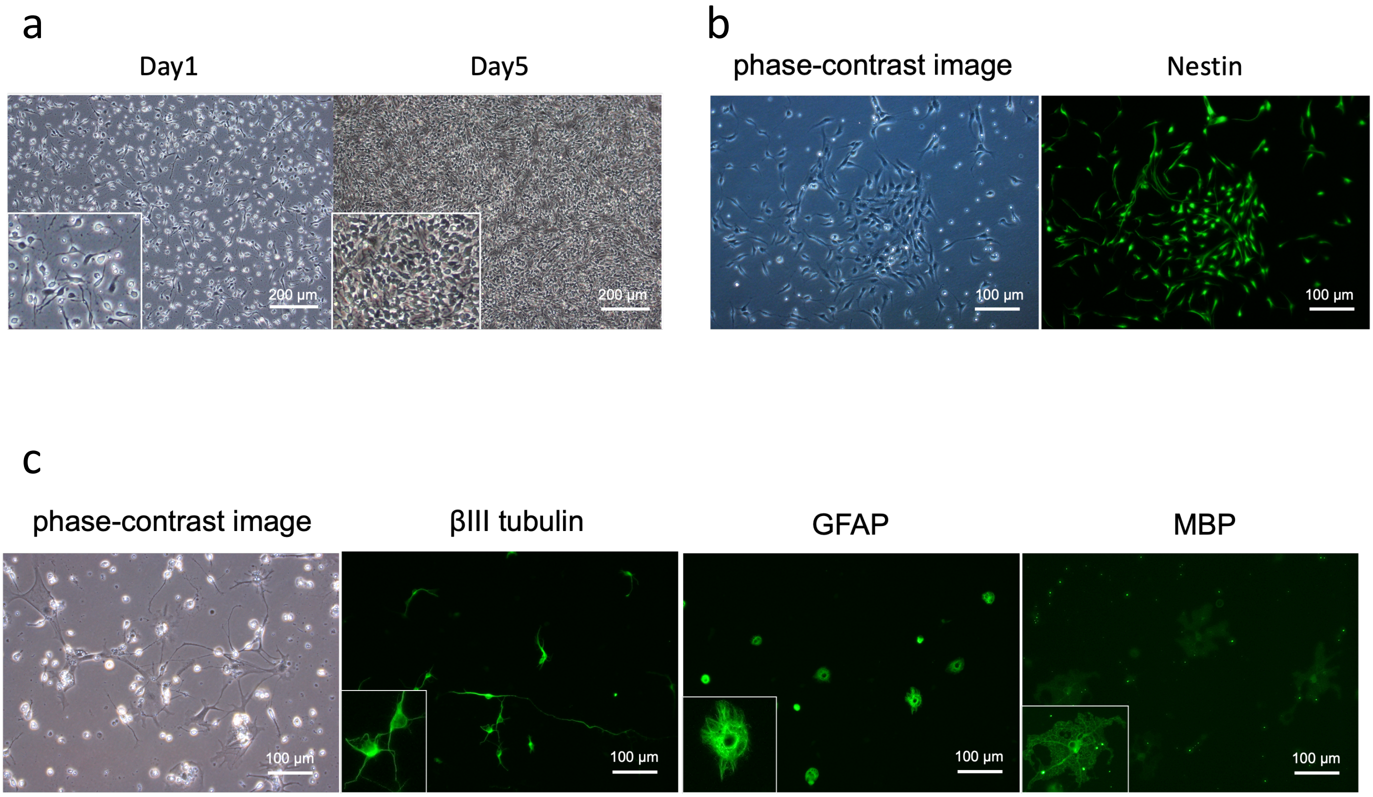

Supplementary Figure 1

Properties as neural stem cells. **a,** Proliferation ability. Phase contrast images of NSCs on Day 1 (Left) and Day 5 (Right) were shown. Cells were cultivated in culture medium with bFGF (10 ng/ml). **b,** Cells express the neural stem cell marker Nestin. **c,** Evaluation of Pluripotency. Immunofluorescence of each differentiation marker, βIII tubulin for neuron, GFAP for astrocyte, and MBP for oligodendrocyte, in cells cultured in bFGF-free culture medium for 1 week were shown.

Supplementary Figure 2

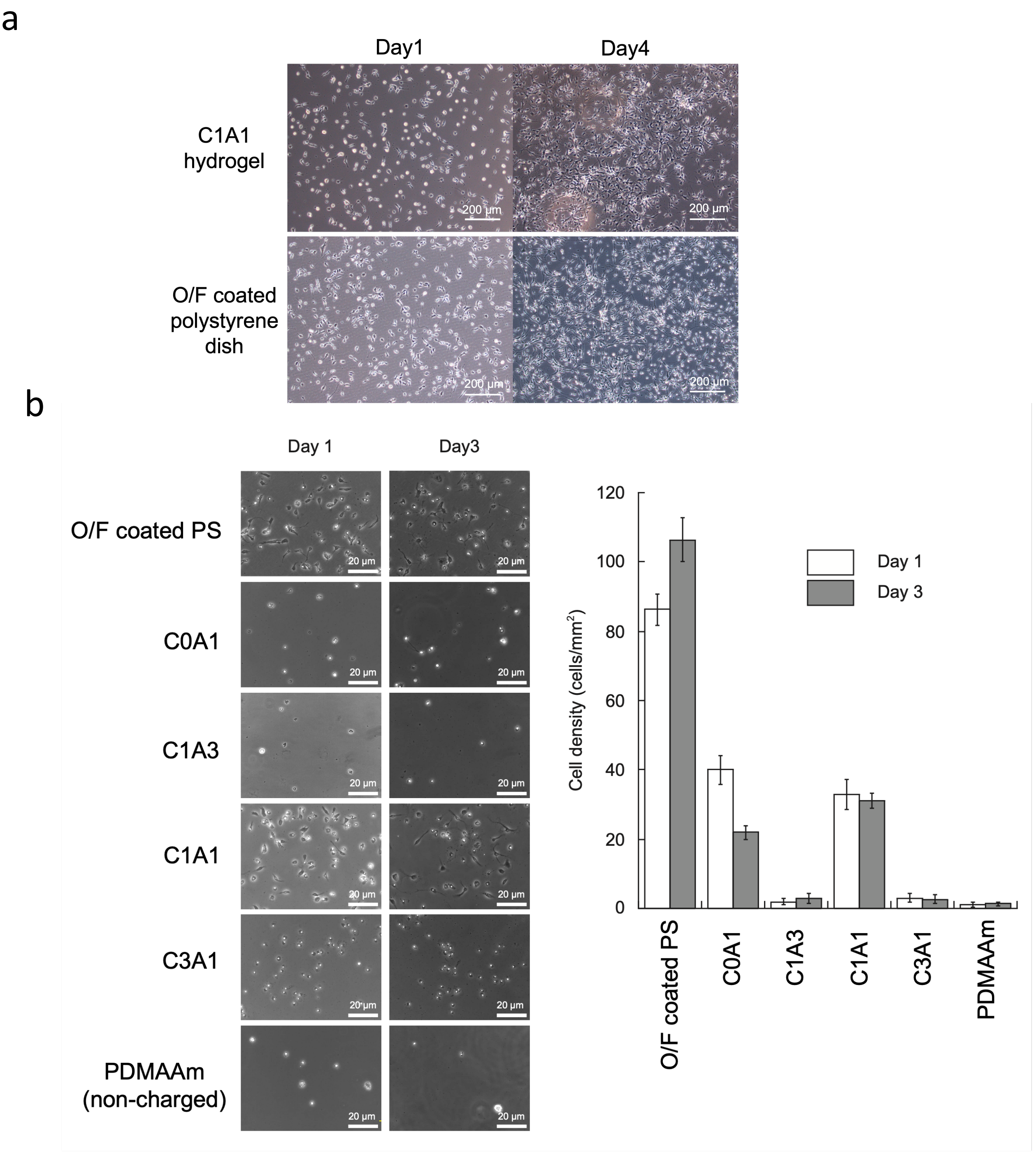

Supplementary Figure 2

Cell proliferation and viability on hydrogel. **a,** Neural stem cells (NSCs) were cultured on C1A1 hydrogel or ornithine-fibronectin (O/F)-coated tissue culture polystyrene (PS) dish, in culture medium supplemented with 10 ng/ml bFGF. After 1 and 4 days, the cells were photographed. **b,** NSCs were seeded at 1×10^4 cells/cm^2 density on O/F-coated PS dish and charged and non-charged hydrogels. After 1 and 3 days, the cells were photographed (left panels), subsequently the surfaces of the PS dish and hydrogels were rinsed with PBS(-), and the cells were dissociated by trypsin-EDTA. Cells were centrifuged at 10,000 $\times$ g for 5 min and suspended with small amount of culture medium. The number of viable cells was counted using hemocytometer after trypan blue staining (Right bar graph).

Supplementary Figure 3

poly (N-(carboxymethyl)-N,N-dimethyl-2-(methacryloyloxy) ethanaminium, inner salt): PCDME

poly(2-acrylamido-2-methyl-propanesulfonic acid): PAMPS

poly(sodium p-styrene sulfonate): PNaSS

poly-dimethylacrylamide: PDMAAm

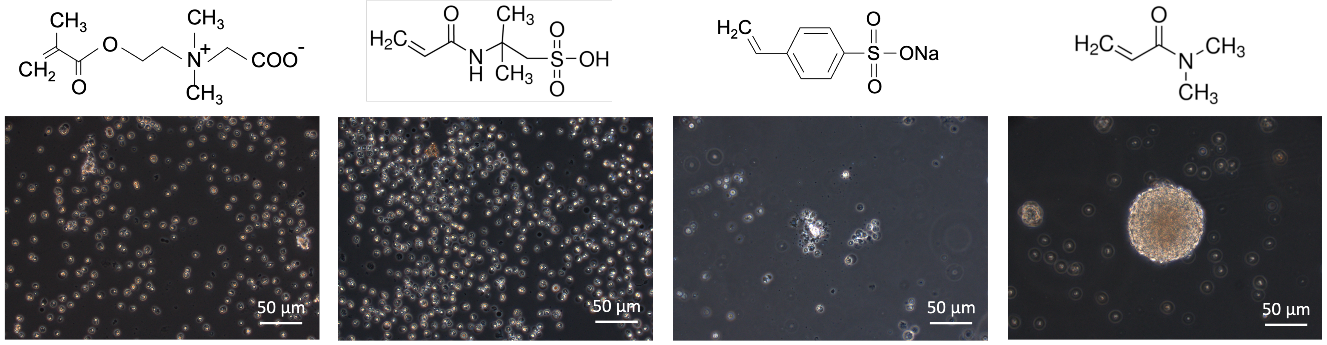

Supplementary Figure 3

Neural stem cell (NSC) cultures on various hydrogel. NSCs were cultured for 3 days on various hydrogels : PCDME, PAMPS, and PNaSS are negatively charged; PDMAAm are neutral hydrogels with no charge. NSCs failed to adhere to any of the hydrogels, indicating unsuitable as scaffolds for the NSCs.

Supplementary Figure 4

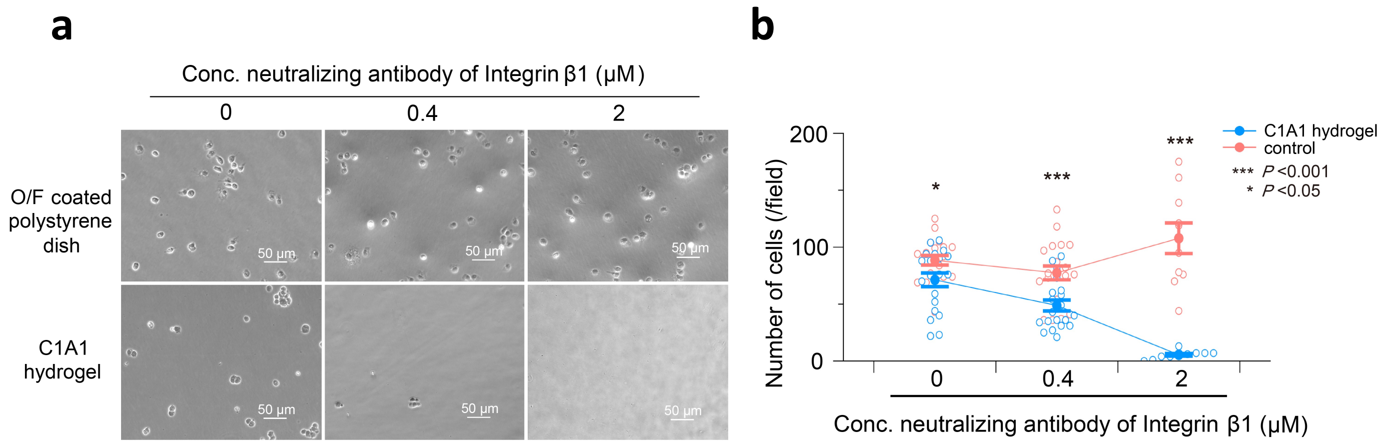

Supplementary Figure 4

A neutralizing antibody against integrin β1 suppressed NSC adhesion to the C1A1 hydrogel. **a,** Phase contrast images of NSCs on the C1A1 hydrogel (lower panels) or poly-L-ornithine/fibronectin-coated polystyrene (PS) dishes as controls (upper panels) are shown. NSCs treated with the indicated concentrations of integrin β1 neutralizing antibodies were seeded onto C1A1 hydrogels or PS dish, and after 1 hour, the cells were photographed. **b,** The numbers of adherent cells were counted in the presence of the indicated concentration of neutralizing antibody and displayed as plots by using the mean ± SEM. ****P* < 0.001. **P* < 0.05.

Supplementary Figure 5

a

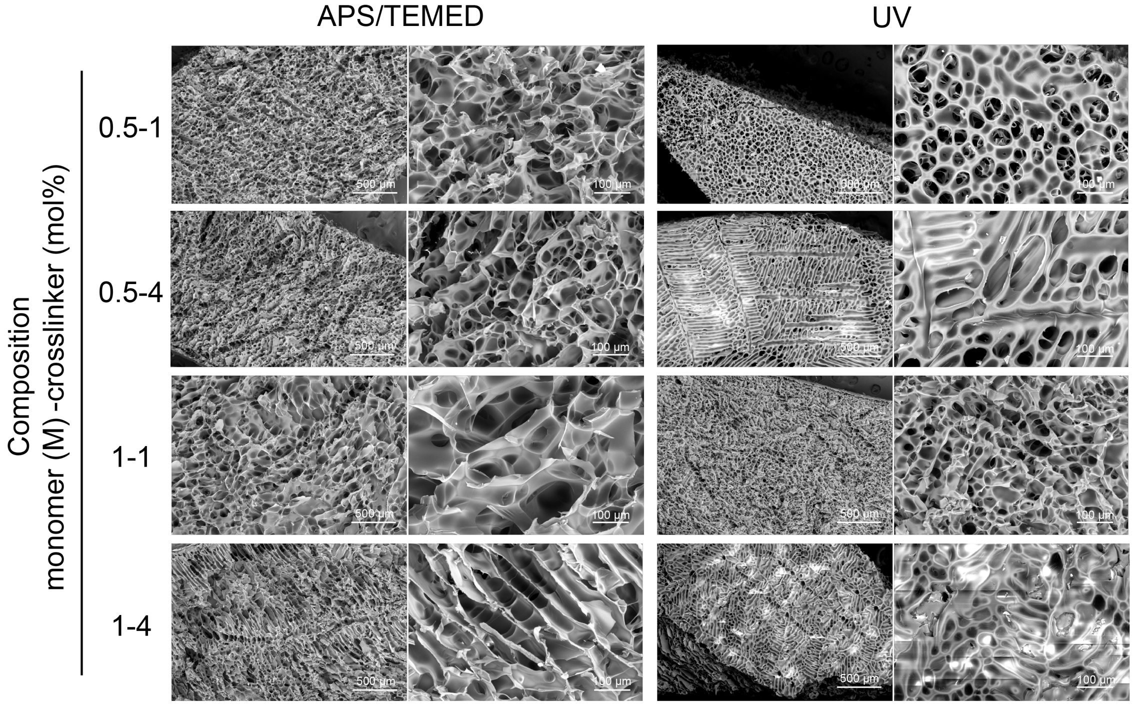

b

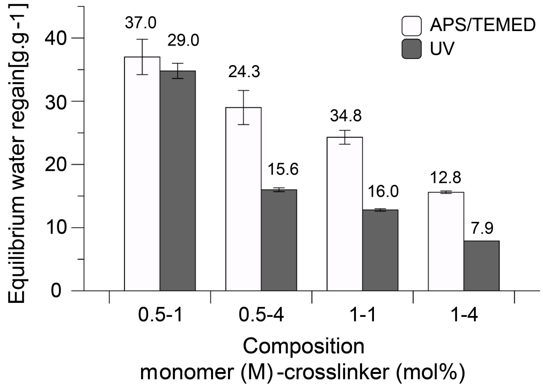

Supplementary Figure 5

Engineering of C1A1 porous hydrogels with different physical properties. C1A1 porous hydrogels were engineered by using four different combinations of monomer and crosslinker concentrations and two polymerization methods: APS/TEMED or UV irradiation. **a,** Scanning electron microscopy images are shown at lower (each left, scale bars, 500 mm) and higher (each right, scale bars, 100 mm) magnifications. **b,** Value of equilibrium water regain for C1A1 porous hydrogels under each condition are displayed as a bar graph using the mean ± SEM. Open bars and closed bars indicate APS/TEMED and UV polymerization, respectively.

Supplementary Figure 6

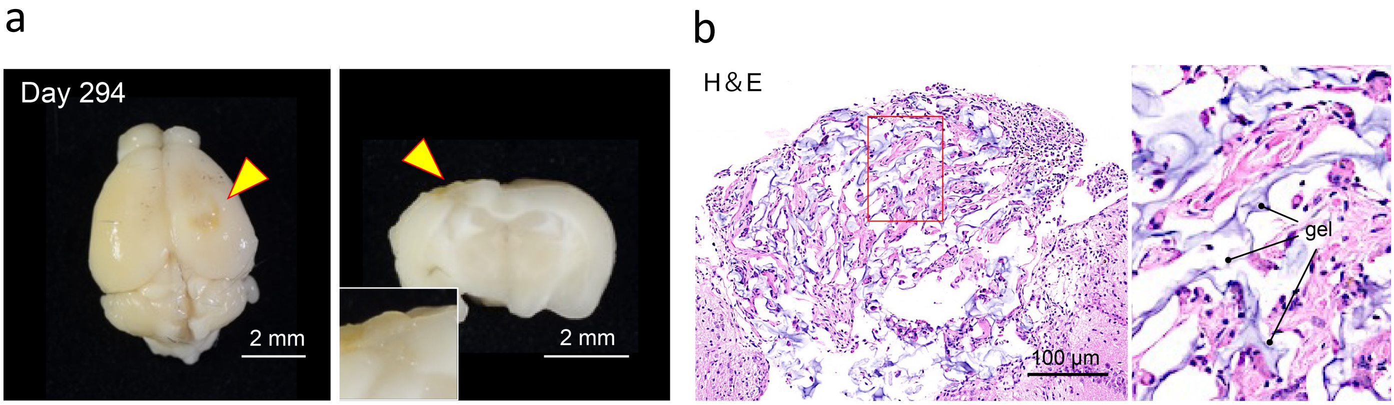

Supplementary Figure 6

Long-term analysis of C1A1 porous hydrogels implanted into mice brains. **a,** Macroscopic photographs of the C1A1 porous hydrogel implanted into mouse brains on day 294. Yellow arrowheads indicate hydrogels. Whole brain (left) and coronal section of the hydrogel-implanted site (right) with a higher magnification (inset of right) are shown. Scale bars, 2 mm. **b,** H & E image of the transplant site. The porous structure of the C1A1 porous hydrogel is shown in light purple. Scale bar, 100 µm.

Supplementary Figure 7

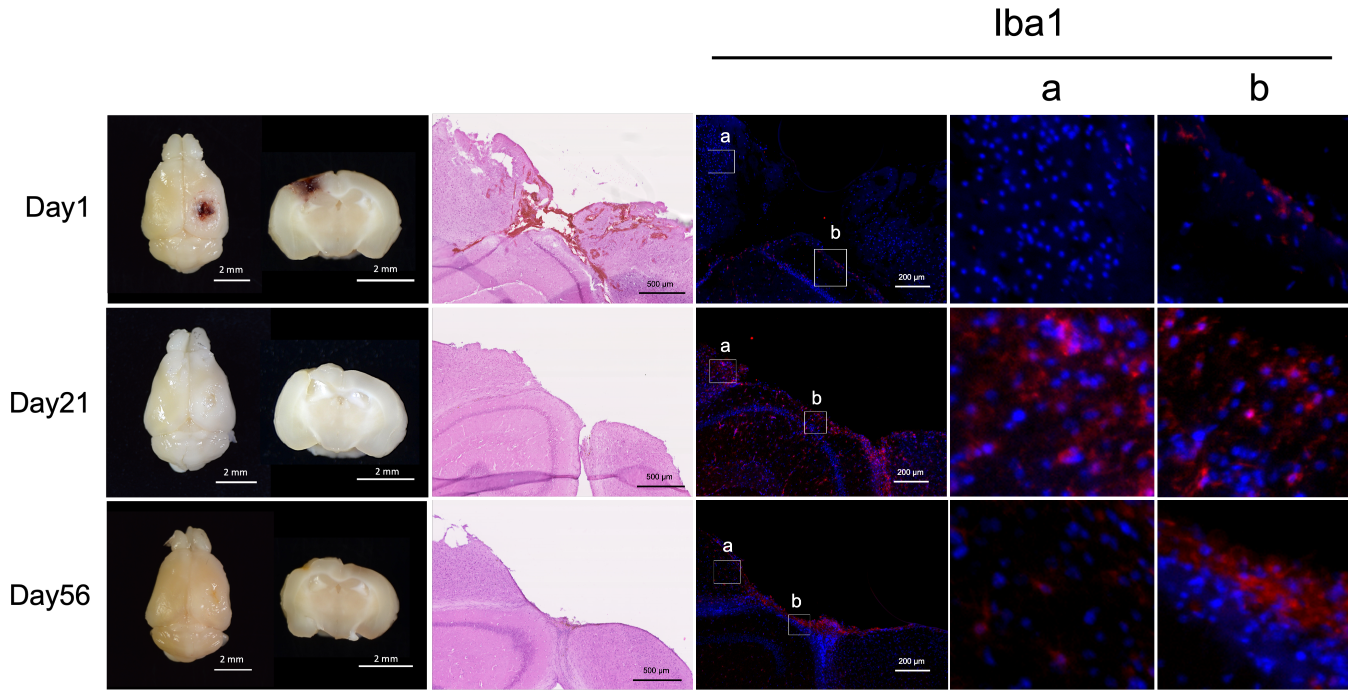

Supplementary Figure 7

Evaluation of tissue reaction around brain cavity. Macroscopic photographs of mice brain (left panels,), H & E images around the hydrogel-implanted site (second panels), and Iba1 immunofluorescence images are shown. “a” is a magnified image of the terminal cortical area and “b“ is a magnified image of the hippocampal surface. On Day 1, hemorrhage remains and only a few Iba1-positive cells appear; on Day 21, hemorrhage dissappeared and many Iba1-positive cells were observed both in the terminal cortex and the hippocampus; on Day56, Iba1-positive cells in the hippocampus and cortex decrease and are observed only in the peri-Cavity area.

Supplementary Figure 8

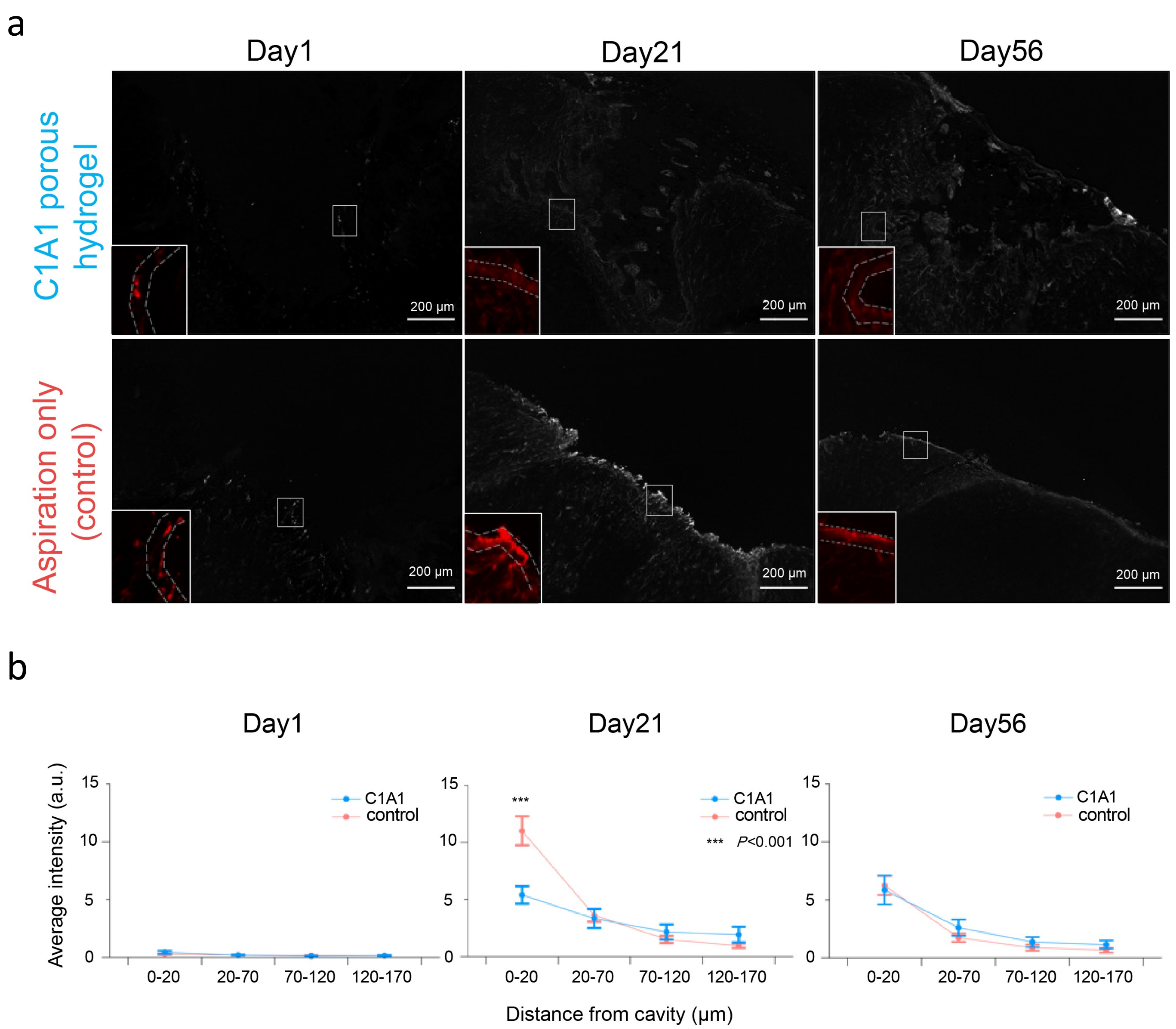

Supplementary Figure 8

Analysis of GFAP-positive cells at the border of brain defects. **a,** Immunofluorescence (IF) images of GFAP-positive cells at the border of brain defects with (upper panels) or without (lower panels) implantation of the C1A1 porous hydrogel on days 1, 21, and 56. Scale bars, 200 µm. The insets show a higher magnification. **b,** The fluorescence intensities of GFAP were measured and displayed as the mean ± SEM. The immunofluorescence images were converted to 8-bit format, and the fluorescence intensities in the regions 0-20 µm, 20-70 µm, 70-120 µm, and 120-170 mm from the boundary were measured. ***P < 0.001.

Supplementary Figure 9

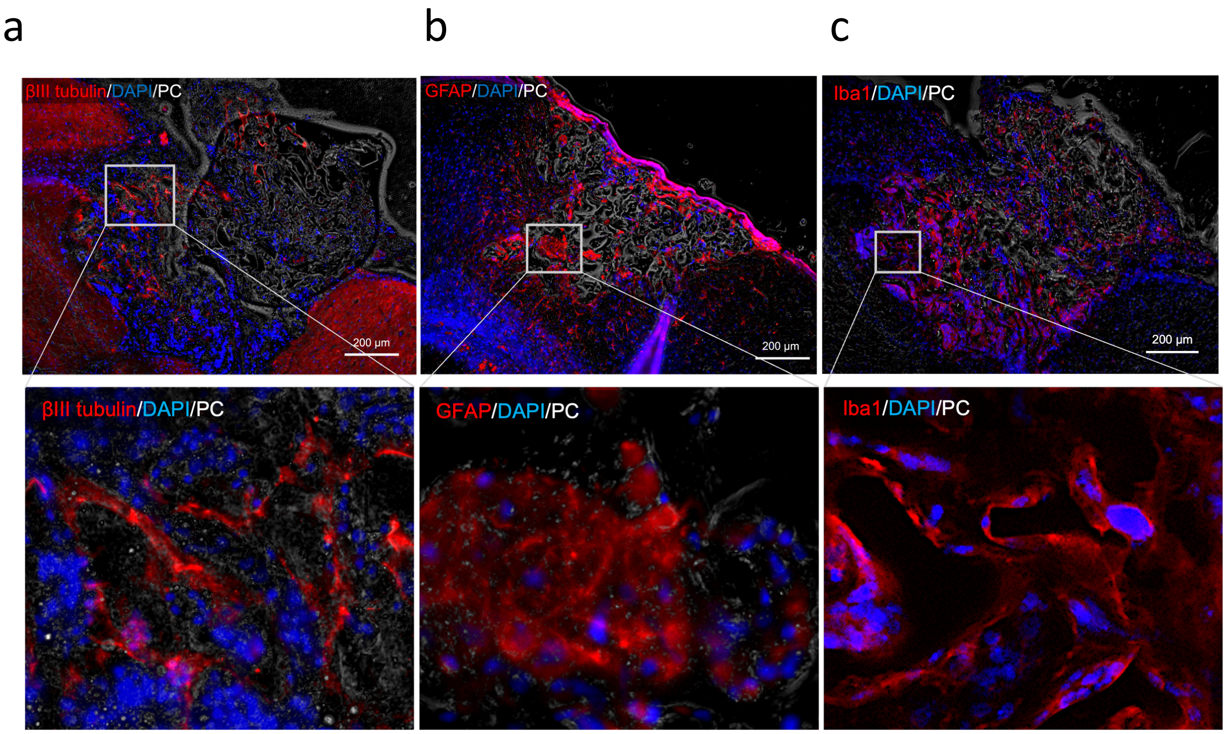

Supplementary Figure 9
Immunofluorescence images of mice brain implanted C1A1 cryogel on Day 56. Immunofluorescence images of βIII tubulin (**a**), GFAP (**b**) and Iba1 (**c**). The lower photos show the enlarged images of upper insets. Scale bars, 200 µm.

Supplementary Figure 10

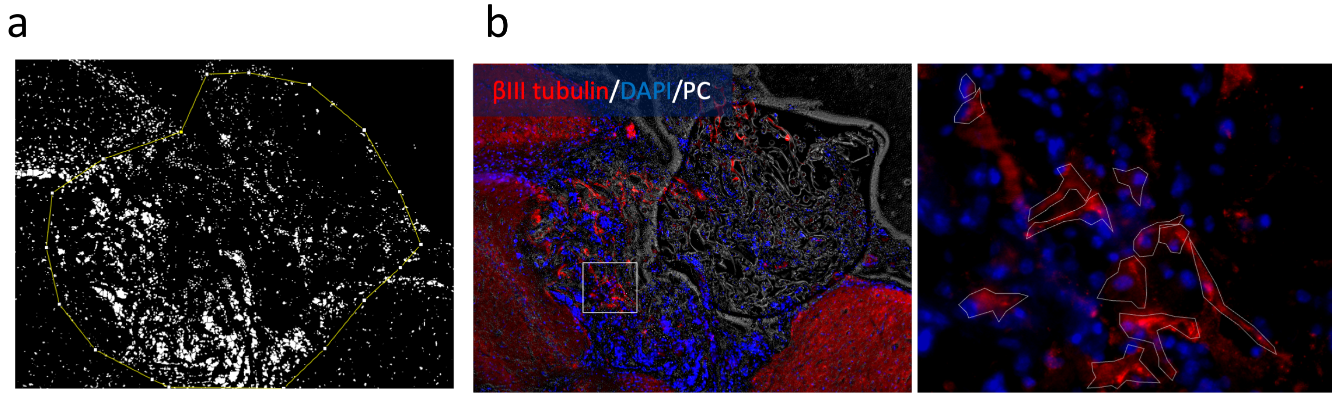

Supplementary Figure 10
Methods of cell count in C1A1 porous hydrogel. Total number of cells in cryogel was measured from two non-contiguous sections staining for DAPI. **a,** DAPI-stained images taken with a 10× objective lens were converted to 8-bit, and DAPI-positive area were masked using a specific threshold value; after processing in Watershed, ROI regions were specified and the number of cells (DAPI+) in the ROI region was measured using Analyze particle. **b,** The number of positive cells for each antibody was determined by immunostaining one section of each case, combining it with DAPI staining, and counting the number of cells.

Supplementary Figure 11

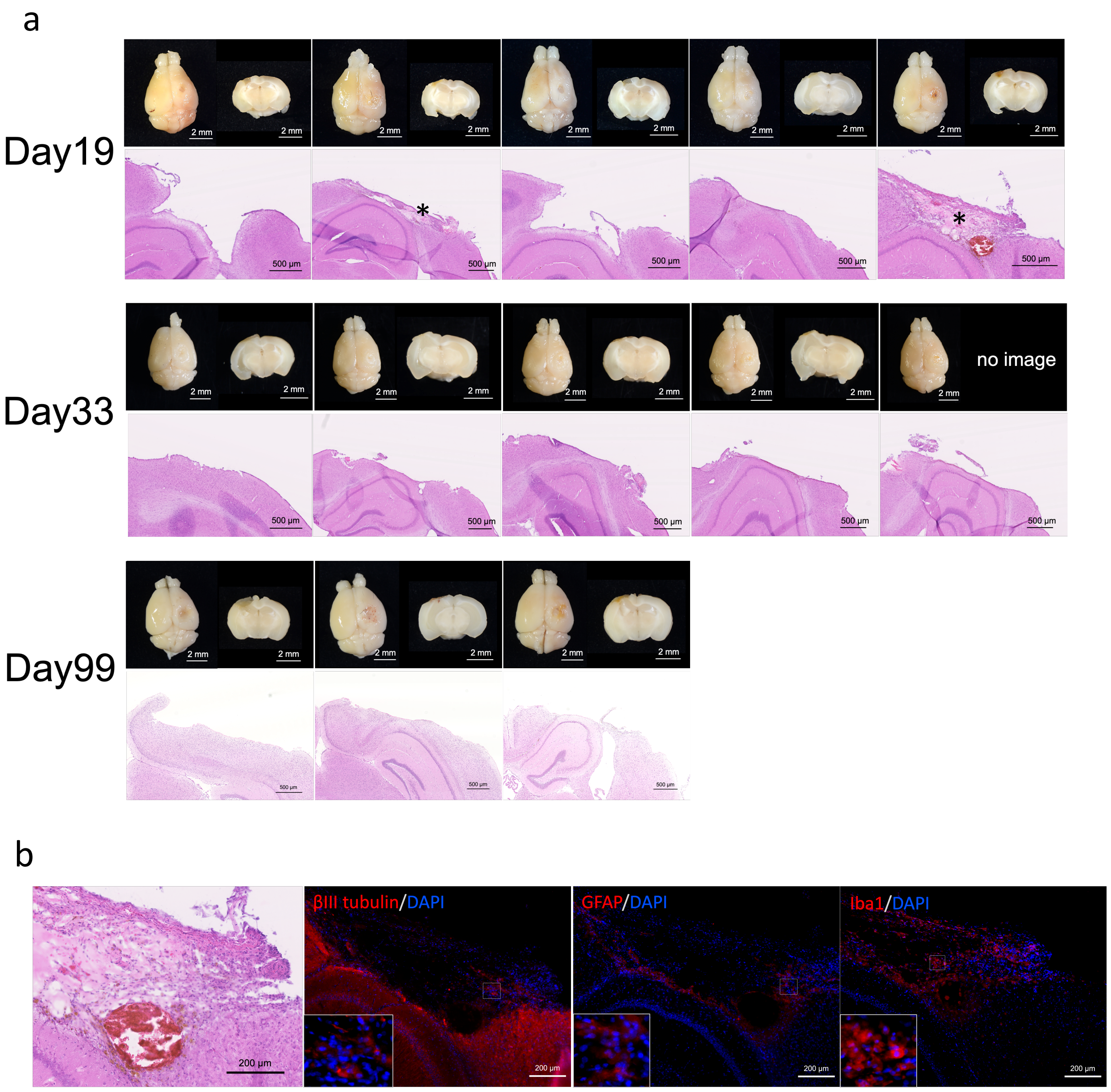

Supplementary Figure 11

Analysis of all 13 mice brains implanted with Matrigel. **a,** Gross and H & E images of samples sacrificed on day 19 (n = 5), day 33 (n = 5) and day 99 (n = 3) after brain resection and Matrigel implantation. *, Matrigel remained in 2 out of 5 mice on day 19. Matrigel disappeared in all samples on day 33 and 99. Samples on day 33 were also transplanted NSCs with Matrigel (These samples were also showed in Figure 6d).

b, Immunofluorescence analysis for βIII tubulin, GFAP, and IbaI in a sample in which Matrigel remained on day 19. H & E image is also shown (left). Scale bar, 200 μm.

Supplementary Figure 12

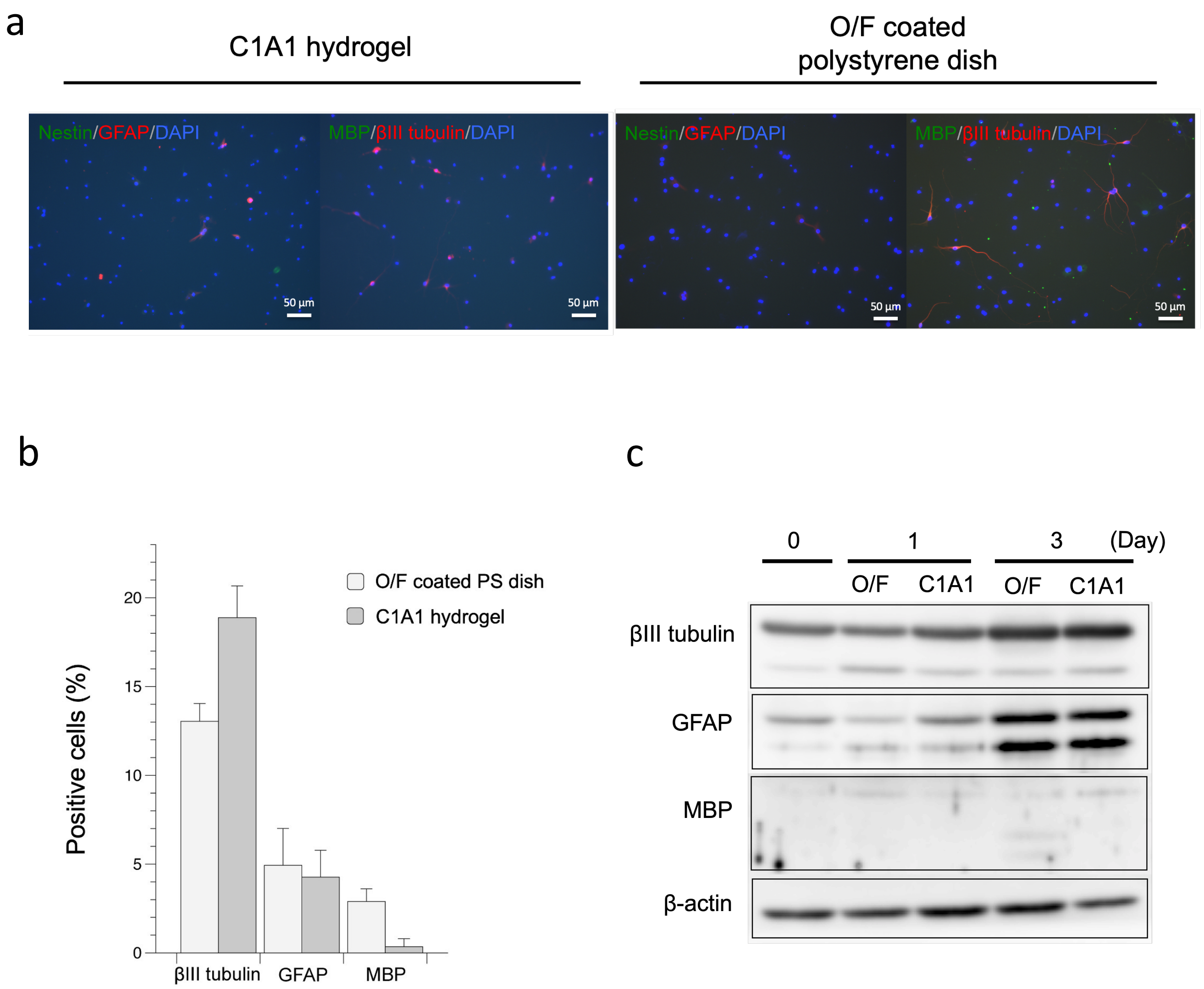

Supplementary Figure 12
Differentiation potential of neural stem cells (NSCs) on C1A1 hydrogel and O/F-coated polystyrene (PS) dish. NSCs were cultivated on the C1A1 hydrogel and O/F-coated PS dish in bFGF-free culture medium for 7 days. **a,** Immunofluorescence images of Nestin (green), GFAP (red), MBP (green), and βIII tubulin (red) are shown. Nuclei were stained with DAPI (blue). **b** The rate of positive cells of each differentiation marker on the C1A1 hydrogel and O/F-coated PS dish are displayed in a bar graph. **c,** Western blotting data of indicated proteins are shown. NSCs were cultured on O/F-coated PS dish and C1A1 hydrogel for 0, 1 and 3 days. β-actin are used as a loading control.

Supplementary Figure 13

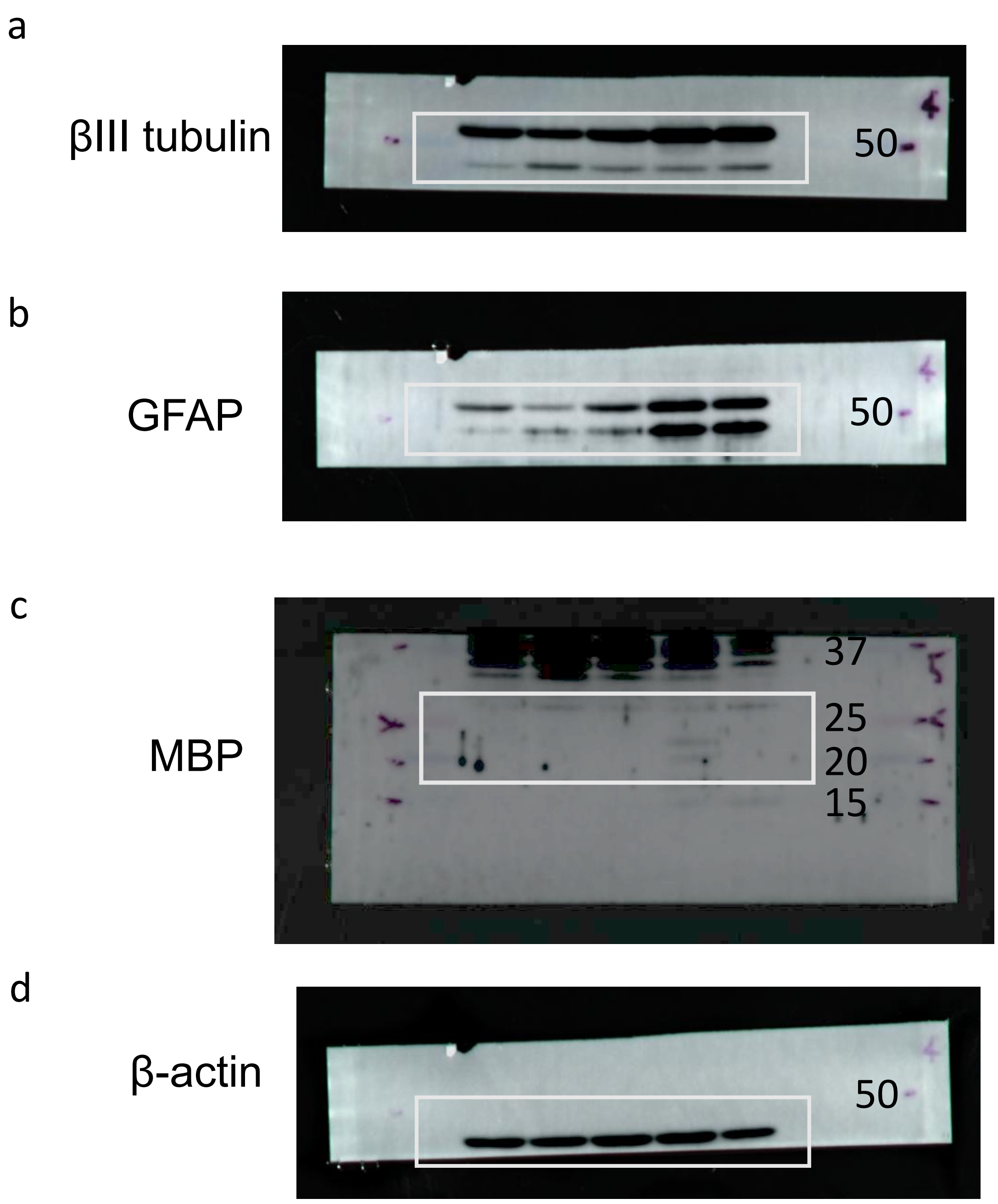

Supplementary Figure 13
Full length membrane data of immunoblotting for Supplementary Fig. 12c with membrane edges visible. **a** βIII tubulin (55 kDa), **b** GFAP (50 kDa), **c** Myelin basic protein (21 kDa and 17 kDa) and **d** β-actin (42 kDa). The membranes for all were cut prior to hybridization with each antibody. a, b, and c were performed stripping and reused with identical membranes. All images were taken with auto exposure time of a LAS4000 mini (GE Healthcare) and automatically merged with a picture of the membrane by LAS4000 mini.

Supplementary movie 1
Three-dimensional image of the C1A1 porous hydrogel.

Supplementary movie 2
In vivo live imaging of vascularization in the C1A1 porous hydrogel.

Supplementary movie 3
Three-dimensional culture of neural stem cells using C1A1 porous hydrogel. Red for βIII tubulin and green for myelin basic protein.

Supplementary movie 4
Three-dimensional culture of neural stem cells using C1A1 porous hydrogel. Red for βIII tubulin and green for myelin basic protein. Blue indicates nuclei visualized by DAPI staining.
